# Targeting Glucose Metabolism to Overcome Resistance to Anticancer Chemotherapy in Breast Cancer

**DOI:** 10.3390/cancers12082252

**Published:** 2020-08-12

**Authors:** Elizabeth Varghese, Samson Mathews Samuel, Alena Líšková, Marek Samec, Peter Kubatka, Dietrich Büsselberg

**Affiliations:** 1 Department of Physiology and Biophysics, Weill Cornell Medicine-Qatar, Education City, Qatar Foundation, Doha 24144, Qatar; elv2007@qatar-med.cornell.edu (E.V.); sms2016@qatar-med.cornell.edu (S.M.S.); 2 Department of Obstetrics and Gynecology, Jessenius Faculty of Medicine, Comenius University in Bratislava, 03601 Martin, Slovakia; liskova80@uniba.sk (A.L.); marek.samec@gmail.com (M.S.); 3 Department of Medical Biology, Jessenius Faculty of Medicine, Comenius University in Bratislava, 03601 Martin, Slovakia; peter.kubatka@uniba.sk

**Keywords:** anticancer drug, breast cancer, cancer, chemoresistance, glucose metabolism, resistance, sensitization, triple-negative breast cancer

## Abstract

Breast cancer (BC) is the most prevalent cancer in women. BC is heterogeneous, with distinct phenotypical and morphological characteristics. These are based on their gene expression profiles, which divide BC into different subtypes, among which the triple-negative breast cancer (TNBC) subtype is the most aggressive one. The growing interest in tumor metabolism emphasizes the role of altered glucose metabolism in driving cancer progression, response to cancer treatment, and its distinct role in therapy resistance. Alterations in glucose metabolism are characterized by increased uptake of glucose, hyperactivated glycolysis, decreased oxidative phosphorylation (OXPHOS) component, and the accumulation of lactate. These deviations are attributed to the upregulation of key glycolytic enzymes and transporters of the glucose metabolic pathway. Key glycolytic enzymes such as hexokinase, lactate dehydrogenase, and enolase are upregulated, thereby conferring resistance towards drugs such as cisplatin, paclitaxel, tamoxifen, and doxorubicin. Besides, drug efflux and detoxification are two energy-dependent mechanisms contributing to resistance. The emergence of resistance to chemotherapy can occur at an early or later stage of the treatment, thus limiting the success and outcome of the therapy. Therefore, understanding the aberrant glucose metabolism in tumors and its link in conferring therapy resistance is essential. Using combinatory treatment with metabolic inhibitors, for example, 2-deoxy-D-glucose (2-DG) and metformin, showed promising results in countering therapy resistance. Newer drug designs such as drugs conjugated to sugars or peptides that utilize the enhanced expression of tumor cell glucose transporters offer selective and efficient drug delivery to cancer cells with less toxicity to healthy cells. Last but not least, naturally occurring compounds of plants defined as phytochemicals manifest a promising approach for the eradication of cancer cells via suppression of essential enzymes or other compartments associated with glycolysis. Their benefits for human health open new opportunities in therapeutic intervention, either alone or in combination with chemotherapeutic drugs. Importantly, phytochemicals as efficacious instruments of anticancer therapy can suppress events leading to chemoresistance of cancer cells. Here, we review the current knowledge of altered glucose metabolism in contributing to resistance to classical anticancer drugs in BC treatment and various ways to target the aberrant metabolism that will serve as a promising strategy for chemosensitizing tumors and overcoming resistance in BC.

## 1. Introduction

Breast cancer (BC) is the most prevalent type of cancer seen in women. Worldwide statistics show that in 2018 approximately two million new cases were detected, with total BC accounting for 11.6% of all cancers [1,2], even though various awareness programs and modern diagnostics increased detection at an early stage. Unfortunately, the majority of BC cases are still detected in later stages as the early stages are often asymptomatic; patients often ignore telltale signs of the disease and avoid screening programs. Depending upon the severity of cancer, treatment modalities include surgery, radiation therapy, chemotherapy, immunotherapy, and targeted therapy [3].

Chemotherapeutic drugs are administered to patients before surgery (as neoadjuvant therapy) or post-surgery (as adjuvant therapy) and are primarily aimed at selective inhibiting the proliferation and activating apoptosis in cancer cells. However, very often, patients encounter moderate to severe side effects in addition to developing acquired resistance to the drug (which is a more adaptive response to the presence of drug) due to prolonged treatment [4,5]. In addition to acquired drug resistance, several other factors contribute to treatment resistance, and variations in the pattern of resistance are observed between different cancers and anticancer drugs [6]. In BC, the response to chemotherapeutic drugs varies with the different BC-subtypes, which, to a certain degree, is attributed to their heterogeneity. Additionally, specific genetic, epigenetic, and metabolic attributes of the subtypes confer an in-built resistance (intrinsic resistance due to gene mutations) against certain drugs [7]. Therefore, changes in the tumor environment, the methylation status of genes, and altered tumor metabolism continually churns out a new subset of cells that keep the tumor advancing in growth and spread [7].

The study of the metabolic characteristics of the tumor and their link to cancer progression is an emerging field in cancer research. Metabolic plasticity in tumors is not only contributed by glycolytic phenotype (as explained by Warburg), but recently mitochondrial energy reprogramming has been identified as a characteristic of the tumor to meet the high demand for energy and biomolecule precursors for rapid cancer cell proliferation. Therefore, recent research emphasizes a hybrid glycolysis/OXPHOS (oxidative phosphorylation) phenotype, rather than a phenotype overly dependent on glycolysis for the energy needs of the cell, thereby significantly contributing to aggressiveness and therapy resistance [8]. The regulation of tumor metabolism as a therapeutic strategy holds promise for any type of cancer, including BC. Vital differences/alterations in glucose metabolism exist between the sensitive and resistant phenotypes of BC [9]. Cancer cells rely on higher rates of aerobic glycolysis as their primary source of energy, even in the presence of oxygen (known as Warburg effect) [10], thus promoting cancer cell proliferation, cancer progression, and resistance to apoptotic cell death [11]. This aberrant tumor glucose metabolism and the increased rates of glycolysis in tumors are correlated to intrinsic/acquired resistance to routinely used anticancer drugs [11,12,13]. In this review, we focus on various ways to target the aberrant metabolism that should serve as a promising strategy for chemosensitizing tumors and overcoming resistance in BC, thus effectively treating BC with minimal side-effects and blocking invasion, metastasis, and incidence of recurrence in affected individuals.

## 2. Altered Glucose Metabolism in Different Subtypes of BC

Originally, BC was broadly classified into hormone receptor-positive BC and hormone receptor-negative, but with the advancement in diagnostics and molecular biology, a more complex and heterogeneous nature of BC was revealed [14]. Heterogeneity varies from patient to patient (inter-tumor) or exists as heterogeneity within the tumor itself (intra-tumor) [15]. The latter defines differences in the cellular, molecular, and metabolic characteristics of the tumor. Identifying molecular subtypes is a major step towards the selection of the treatment strategy and prediction of the treatment outcome. Based on the presence or absence of the estrogen receptor (ER), progesterone receptor (PR), human epidermal growth factor receptor 2 (HER2), and Ki-67 status, BCs are classified as HER2+ (ER−,PR−, HER2 overexpressed), luminal A (ER+,PR+, HER2−, Ki 67 low), luminal B (ER+, PR low, HER2−, Ki 67 high), and basal/triple-negative (ER−,PR−, HER2−) subtypes [16]. Invasive BC, which is positive for either one or more of the three important biomarkers ER, PR, and HER2, can be targeted by hormone-based therapy, whereas tumors that are negative for all three receptors, classified as triple-negative breast cancers (TNBC), do not benefit from hormone-based therapy. Six subtypes of TNBCs were identified using gene expression analysis: Basal-like 1 (BL1), Basal-like 2 (BL2), Mesenchymal (M), Mesenchymal stem-like (MSL), immunomodulatory (IM), and luminal androgen receptor (LAR). This reveals the complexity of the treatment of TNBC [14,17,18]. Hitherto, there are no effective targeted drugs identified, and systemic treatment with cytotoxic agents remains the standard therapeutic strategy.

Identifying metabolic differences among BC molecular subtypes helps to fine-tune strategies to target specific enzymes and transporters. Metabolomic studies expand the scope of tumor classification beyond the present molecular subtypes and add value to clinical trials. Cappelletti V. et al. [19] reported metabolic differences among BC subtypes in patients with primary BC undergoing surgery. Significant differences in the enzyme activity and pathways were reported for glucose metabolism [19]. The higher glycolytic potential is frequently linked to therapeutic resistance [20]. There were considerable metabolic differences among HER2+, luminal A, and luminal B subtypes [21]. Perturbations in glucose metabolism were more prominent in the HER2+ BC and the luminal B subtype. Although the level of glucose utilization was the same among the three subtypes, the accumulation of metabolites in the downstream glycolytic pathway was higher in luminal B, basal-like, and HER2+ than in luminal A [19].

The increased reliance of cancer cells on aerobic glycolysis, although less efficient in generating ATP, not only meets the energy requirements of the rapidly proliferating cancer cells, but also suffices the higher demand for metabolic intermediates required for anabolic reactions [22,23]. Glycolytic intermediates form the carbon backbone for the biosynthesis of nucleotides, amino acids, and lipids [22,23]. The glycolytic intermediate glucose-6-phosphate is channeled to the pentose phosphate pathway and converted to ribose-5-phosphate, which is then used for nucleotide biosynthesis [23,24]. The dihydroxyacetone phosphate (DHAP), another intermediate in the glycolytic pathway, fuels the de novo synthesis of lipid in addition to the mitochondrial tricarboxylic acid cycle (TCA) intermediates, such as acetyl coenzyme A (Acetyl CoA) which further supports lipid and cholesterol synthesis [22,24]. Cellular lipogenesis is essential for membrane biogenesis and hence high rate of lipogenesis is indicated in rapidly proliferating tumor cells [25]. Additionally, the glycolytic intermediate 3-phosphogycerate is a precursor for amino acid and nucleotide biosynthetic pathways [24,26]. Looking at the metabolite status from the clinical point of view a significant difference in the lactate levels was observed with basal-like subtype having more lactate than luminal A subtype. Similarly, high levels of fructose 1,6 bisphosphate (F1,6BP) were reported in the basal-like subtype of BC. Ribulose 5-phosphate and xylulose 5-phosphate of the pentose phosphate pathway (PPP) are accumulated in HER2+ BC [19]. Analysis of the TCA metabolites did not show significant differences, except for the fumarate and malate levels [19]. These metabolites can fuel fatty acid biosynthesis and cholesterol synthesis, thereby promoting tumor growth [27]. Abnormally high levels of fumarate and succinate were showed to stabilize hypoxia inducible factor 1-alpha (HIF-1α) and related genes and created a pseudo-hypoxic condition in tumors promoting angiogenesis, migration, and metastasis [28]. Moreover, these metabolites change the epigenetic profile of the cells as well [28]. Similarly, 3-phosphoglycerate, a glycolytic intermediate, fuels the de novo biosynthesis of serine and one of the enzymes, phosphoglycerate dehydrogenase (PHGDH), which catalyzes this process and is highly expressed in TNBCs [28]. An in-depth review by Amelio I. and co-workers [29] mentions serine as an allosteric modulator of glycolytic enzyme pyruvate kinase M2 isoform (PKM2). High serine levels can fully activate the PKM2 enzyme driving the cell to a more aerobic glycolysis process and lactate production. Conversely, upon serine depletion, PKM2 activity is reduced, as a result the cells switch to mitochondrial mode of metabolism. Serine is also linked to other metabolic pathways such as glycine metabolism, which has a significant role in tumorigenesis [29]. High rates of glycolysis and the associated poor prognosis in BCs were linked to an increased level of lipogenesis, marked by the overexpression of key fatty acid synthesis enzymes such as ATP citrate lyase, acetyl CoA carboxylase, and fatty acid synthase [30,31].

The enzyme succinate dehydrogenase (SDH; composed of the four subunits: SDHA, SDHB, SDHC, and SDHD), catalyzing succinate oxidation in TCA, was differently expressed in BC subtypes. A higher level of SDHA and SDHB was detected in HER-2 subtype. On the contrary, lower or absent expression of SDHA and SDHB was documented in Luminal A and TNBC, respectively. Moreover, it was observed that the lower expression profile of SDH was more frequent among young patients and patients with low-grade histology [32].

Metabolic profiling of TNBC in vitro revealed an altered metabolism that links to cell signaling by epidermal growth factor receptor (EGFR) and receptor tyrosine kinase (RTK) [33]. In the TNBC cell line, MDA-MB-468 overexpressing EGFR showed a high rate of glycolysis upon stimulation with epidermal growth factor (EGF). Increased lactate production was found to show dependency on EGF/EGFR signaling and, in fact, EGFR signaling correlated with T cell inactivity and immune evasion when co-cultured with T cells [33]. Therefore, targeting these signaling pathways could change the metabolic vulnerabilities in TNBC. For example, TNBC is less sensitive to metformin treatment in the presence of high glucose [34].

Similarly, high enolase (EN) and hexokinase (HK) activity are linked to tamoxifen resistance in ER+ BC [35,36]. Additionally, HK2 and phosphofructokinase (PFK-L) status indicate the risk of relapse, aggressiveness, and poor prognosis in BC patients [37,38]. A review by Sun X et al. [39] discusses various glycolytic enzymes and other metabolic pathways dysregulated in TNBC and the metabolic adaptations in metastasis and resistance. Earlier observations are in agreement with proteomic profiling of BC cells, revealed that most of the proteins overexpressed belonged to the glycolytic pathway [40].

## 3. Glycolytic Pathway Targeted by Classical Anticancer Drugs and Other Compounds

**Figure 1:**
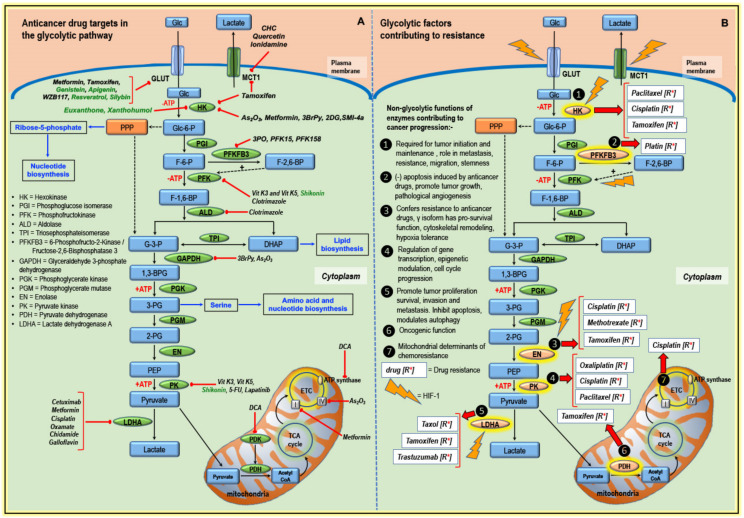
An illustration of glucose metabolism leading from the glycolytic pathway to the tricarboxylic acid cycle (TCA) and electron transport chain (ETC). (**A**) Targets and inhibitors of the glycolytic pathway, TCA, and ETC. These inhibitors target key enzymes/components, thereby regulating glucose metabolism. The bold red lines with rounded ends indicate the inhibitory effect of the various agents on target enzymes/components. Classical anticancer drugs like cisplatin, As_2_O_3_, and doxorubicin can modulate glycolytic enzymes. Some natural compounds (shown in the green color font) such as resveratrol, genistein, and shikonin can modify the activity of these enzymes. The blue arrows represent how metabolites of the glycolytic pathway contribute to other key biosynthetic pathways that support cancer cell survival and growth. The precursors for de novo biosynthesis of nucleotides are derived from ribose 5-phosphate of the pentose phosphate pathway (PPP), which is a side branch of glucose metabolic pathway. Likewise, 3-PG, a glycolytic intermediate, serves as a precursor for amino acid synthesis, thus fueling anabolic reactions necessary for cancer growth. Similarly, an enhanced lipogenesis controlled by an oncogenic signaling is often associated with an increased tumor glycolysis. Here, DHAP acts as an intermediate for lipid biosynthesis. (**B**) The various enzymes/components that contribute to drug resistance in cancers and how the non-glycolytic functions of these enzymes contribute to cancer progression is highlighted. The transcription factor, hypoxia-inducible factor-1 (HIF-1), upregulates the expression of the key transporters and glycolytic enzymes, thereby promoting adaptive cancer cell metabolism and supporting tumor progression. Abbreviations; Glc = glucose, Glc-6-P = glucose-6-phosphate, F-6-P = fructose-6-phosphate, F-1,6-P = fructose 1,6 bisphosphate, G-3-P = glyceraldehyde 3 phosphate, DHAP = dihydroxyacetone phosphate, 1,3-BPG = 1,3-bisphosphoglycerate, 3-PG = 3-phosphoglycerate, 2-PG = 2-phosphoglycerate, PEP = phosphoenolpyruvate, GLUT = glucose transporter, MCT1 = monocarboxylate transporter 1, SMI-4a = (5Z)-5-[[3-(Trifluoromethyl)phenyl]methylene]-2,4-thiazolidinedione, 3PO = 3-(3-pyridinyl)-1-(4-pyridinyl)-2-propen-1-one, 3BrPy = 3-bromopyruvate, 2DG = 2-deoxy-D-glucose, DCA = dichloroacetate.

Enhanced glycolysis correlates with the upregulation and activation of critical glycolytic enzymes, which, in turn, promotes tumorigenesis [41]. Targeting key glycolytic enzymes enables the rewiring of altered tumor metabolism, thereby sensitizing or resensitizing (in case of resistance) tumors to chemotherapy. Many compounds, including natural compounds, influence the tumor microenvironment by regulating HIF-1α expression and can dysregulate glucose metabolism [42,43]. Selected inhibitors of the transporters, glycolysis, and mitochondrial components are illustrated and summarized in Figure 1A and Table 1.

### 3.1. Targeting Transporters

GLUTs, the glucose transporters in mammalian cells, favor aerobic glycolysis [70]. Increased glucose utilization, activated glycolysis driven by oncogenic signaling to derive energy leading to lactate accumulation in cancer cells can occur even in the presence of oxygen. Concerning the glucose transporters (GLUT-1), the expression was higher in TNBC (basal-like) than in non-TNBC [39]. Frequently, GLUT-1 and GLUT-3 are upregulated in BC cell lines MDA-MB-435 and MDA-MB-231 [71]. Constitutively activated HIF-1α in cancer cells is associated with an upregulated of GLUT-1 and GLUT-3 expression [72]. Other studies mentioned that immunohistochemical patterns to 18-fluorodeoxyglucose (18FdG) uptake showed a significant correlation with GLUT-1 expression and HIF-1α [73]. Several compounds have shown their capability to bind to the different domains of GLUT transporters and thus influence the activity and function of the transporters. For example, genistein binds to the exofacial side of GLUT transporter, whereas quercetin, tyrphostin A47, and tyrphostin B46 bind to the cytoplasmic side of the cell (Table 1) [74,75]. A small molecule inhibitor WZB117 and resveratrol can directly inhibit GLUT-1 transporter [76,77], whereas genistein has been reported to be a competitive inhibitor of GLUT-1 transporter, thus affects transport of hexoses and dehydroascorbic acid. Tamoxifen, a specific ER modulator, widely used in BC treatment, possess an anti-estrogenic effect, which in turn reduces the GLUT-1 expression by two-fold in BC cells (Figure 1A) [44]. Therefore, GLUT-1 is used as a marker for treatment response to tamoxifen [44]. Sawayama H. et al. [78] studied the influence of GLUT-1 inhibition on cisplatin response in esophageal cancer and found that the inhibition of GLUT-1 using miRNAs or specific inhibitors improved the sensitivity to low dose cisplatin treatment [44,78]. Metformin, a widely used antidiabetic drug, is also being investigated for its anticancer potential [45]. Metformin can inhibit proliferation and induce apoptosis in BC cells by downregulating all isoforms of GLUT (Figure 1A) [46]. However, metformin is reported to upregulate GLUT-12 in TNBC [46]. Resveratrol, a polyphenol having many bioactivities, has been shown to interact non-competitively with GLUT-1 (Figure 1A) [77]. Furthermore, natural flavonoids, including hesperetin, kaempferol, and epigallocatechin-3-gallate (EGCG), significantly reduced the level of GLUT-1 in BC (4T1, MCF-7, and MDA-MB-231) cell lines [47,48,49].

The monocarboxylate transporter (MCT) is a lactate efflux transporter, which is essential for maintaining pH and for regulating glycolysis. MCT 1–4 were biochemically characterized, and MCT1 and MCT4 are often associated with tumors [79,80]. Therefore, they are attractive targets for anticancer therapy [81]. As observed in HeLa cells, HIF-1α under hypoxic conditions increased the expression of MCT4 but did not have any influence on MCT1 expression. It is observed that the physiological function of MCT4 is to transport lactate out of the glycolytic cell [82]. MCT1 primarily deals with pyruvate export and lactate uptake [83]. Studies report an elevated MCT1 expression in aggressive BC with glycolytic phenotype. Inhibition of MCT1 using blockers (in HS578T, SUM149PT, and SUM159PT BC cells) attenuated pyruvate transport but not lactate. Notably, blocking of MCT1 enhanced the oxidative metabolism and parallelly reduced BC cell proliferation [83]. In a tumor microenvironment, MCT pumps lactate to the extracellular space creating an acidic condition favoring cancer progression. Analysis of MCT1 expression in BC tissue samples indicated the highest expression in TNBC than in ER+, PR+, or HER2+ BC.

Additionally, MCT1 expression correlates with the aggressiveness, recurrence, reduced survival, and tumorigenicity [52,84] in BC. Hyperglycolytic activity due to lactate efflux can be inhibited using natural compounds, siRNA, and small molecule inhibitors (Table 1). Inhibition of MCT1 using inhibitors can have a direct influence on lactate transport and intracellular pH regulation and can control cancer cell viability [53]. Morais-Santos F. et al. [53] observed differences in sensitivity to MCT1 inhibitors among various subtypes of BC. Here, they observed and reported that basal subtypes showed a difference in sensitivity to MCT1 inhibitors such as quercetin, α-cyano-4-hydroxycinnamate (CHC), and lonidamine (Figure 1A). In the case of quercetin, basal type BC (MDA-MB-468, Hs 578T) with high MCT1 expression showed more sensitivity to quercetin than BC with low MCT1. In these cell lines, the concomitant reduction in glucose consumption and lactate production was observed.

Conversely, BT-20 basal type cells were insensitive to all three MCT1 inhibitors, despite high MCT1 expression [53]. This study emphasizes the different mechanisms by which the inhibitors target the receptor and how the difference in sensitivity varies with the metabolic phenotypes in various subtypes of BC. Further research in the area of metabolic phenotypes towards predicting drug sensitivity is warranted to advance in target-based therapy. However, the clinical use of MCT1 inhibitors has been reported to have serious side effects like myopathy [85].

Sodium/glucose co-transporter 1 (SGLT1) is essential for maintaining intracellular glucose concentration and can promote tumor growth by interacting with the epidermal growth factor receptor (EGFR) [86]. In vitro and in vivo analysis of SGTL1 transporter showed an important link between SGLT1-EGFR, regulating TNBC cell survival. mRNA analysis of this transporter in BC tissue samples indicated the highest expression in TNBC and HER2+ than in luminal A or luminal B [86]. The same research group also showed that knockdown of SGLT1 significantly reduced TNBC proliferation and tumor size.

### 3.2. Targeting Enzymes of the Glycolytic Pathway

Hexokinase (HK) catalyzes the first step in glucose metabolism converting glucose to glucose-6-phosphate. There are four significant isoforms of HK, named HK1, HK2, HK3, and HK4, which differ in their cellular distribution and affinity to its substrate glucose. HK1 and HK2 are mitochondrial kinases and associated with AKT-mediated cell survival [87,88]. HK2 status is clinically linked to recurrence and poor prognosis in BC [37]. Clinical studies conducted on BC patients detected 44% positive out of 118 cases for high expression of HK2 [37]. Inhibition of HK2 has shown to inhibit proliferation and change the metabolic profile of cancer by shifting from glycolytic to OXPHOS pathway with reduced lactate formation [89]. 2-Deoxy-D-glucose (2-DG), a glucose analog, competes with glucose for GLUT transporters [90]. As 2-DG cannot be metabolized, it becomes cytotoxic to the cell [91], and HK can modulate 2-DG induced cytotoxicity [55]. A combination of polydatin (a precursor of resveratrol) with 2-DG induced apoptosis, inhibited metastasis, and invasion in MCF-7 cells via HIF-1α/HK2 axis [92]. In vitro studies showed that metformin could directly inhibit HK2 activity in MDA-MB-231 cells and inhibit tumor growth in vivo (Figure 1A) [54]. Interestingly, metformin showed selective inhibition of HK1 and HK2 isoforms. Parallelly, metformin decreased the glycolytic rate and activated the 5′ adenosine monophosphate-activated protein kinase (AMPK) pathway. Similarly, in another study, co-treatment with 2-DG and metformin in MDA-MB-231 cells caused the detachment of cells and activation of AMPK signaling [93]. 3-Bromopyruvate (3BrPy) is a potent inhibitor of glyceraldehyde-3-phosphate dehydrogenase (GAPDH) and HK2 and can be selectively taken up into the cell via the highly expressed MCT1 lactate transporter in tumor cells (Figure 1A) [58]. The advantage of facilitated drug delivery is that healthy cells can be spared, and only tumor cells tend to accumulate the drug, thus minimizing the side effects.

6-Phosphofructo-2-kinase/fructose-2,6-bisphosphatase-3 (PFKFB3) is an enzyme that controls the level of fructose-2,6-bisphosphate and is crucial in BC cancer progression [94]. Blocking PFKFB3 by 3-(3-pyridinyl)-1-(4-pyridinyl)-2-propen-1-one (3PO) can negatively regulate glycolysis and modulate rat sarcoma viral proto-oncogene (Ras) and protein kinase B (AKT) in HER2+ BC cells [56]. Inhibitors of PFKFB3 can target specific cell types such as cancer cells, cancer stem cells, endothelial cells, and immune cells [57]. Perhaps due to the ubiquitous nature of GAPDH, considering potential side effects when targeting it, the scope for developing GAPDH as an anticancer target has diverse opinions among the scientists [95]. Besides, several studies identified the prominent role of Lactate dehydrogenase A (LDHA) in TNBCs [39,96,97]. Oxamate, a competitive inhibitor of LDHA is a pyruvate analog that inhibited glycolysis and decreased the viability in both paclitaxel sensitive and resistant MDA-MB-435 cells (Figure 1A) [62]. Galloflavin, another LDHA inhibitor, showed two different mechanisms of inhibition of cancer cell proliferation (Figure 1A). In MCF-7 cells, the inhibition of proliferation by galloflavin correlated to the downregulation of cell survival pathway. Galloflavin induced oxidative stress that, in turn, suppressed cell proliferation of TNBC (MDA-MB-231) cells [98]. Certain compounds, such as chidamide, inhibited glycolysis via a miRNA-33a-5p-mediated inhibition of LDHA in MDA-MB-231 and BT20 cells (Figure 1A) [64]. It is important to note that several flavonoids manifested anticancer potential via inhibition of the glycolytic enzymes. EGCG suppressed mRNA levels and activities of glycolysis-related enzymes, including HK2, PFK, LDH, and PKM2, in 4T1 cells [49]. Additionally, quercetin-regulated glucose uptake and the production of lactic acid reduced the level of PKM2 and LDHA in MCF-7 and MDA-MB-231 cells [99]. An interesting phenomenon represents a binding between the mitochondria membrane and HK2. An interaction between voltage-dependent anion channels (VDACs) located in the outer mitochondria membrane and HK2 is tightly associated with resistance to apoptosis. Moreover, recent evidence revealed that HK2/VDAC interaction led to the development of drug resistance. An essential role of HK2 in resistance to gemcitabine (GEM)-induced apoptosis was demonstrated in pancreatic cancer. It has been observed that HK2 dimerization promoted interaction with VDACs, resulting in inhibition of GEM-induced apoptosis [100]. One possibility to circumvent HK2/VDACs interaction represents the application of synthetic or natural drugs able to disrupt mitochondrial binding of HK2. Recently, Guo Y. et al. [101] used synthesized flavonoid GL-V9 against BC MCF-7 and MDA-MB-231 cell lines. Obtained data showed that GL-V9 induced dissociation of HK2 from VDAC, leading to inhibition of glycolysis and mitochondrial-mediated apoptosis. Similarly, another synthesized flavonoid FV-429 disrupted the interaction between HK2 and VDAC in MDA-MB-231, resulting in cell death [102]. Targeting HK2/VDAC interaction thus represents a promising approach to overcome drug resistance and inducing apoptosis in cancer cells. In summary, beneficial properties of phytochemicals can act as powerful tools against tumor development via modulation of cancer cells metabolism, and their combination with classical anticancer drugs brings new opportunities in the therapeutic strategy of the 21st century.

### 3.3. Targeting Components of Mitochondria

While discussing the dependence of cancer cells on aerobic glycolysis, it is essential to highlight the metabolic flexibility of cancer under hypoxic and normoxic conditions. Tumors become increasingly hypoxic with an increase in distance from the blood vessel, and those cancer cells nearer to the blood vessel will be better oxygenated; therefore, the region will bear a normoxic signature [103]. Moreover, there is a mutual association between stromal cells and cancer cells for metabolic purposes in the tumor. Cancer cells induce stromal cells to rely on aerobic glycolysis, and the metabolites accumulated by stromal cells are used by cancer cells for mitochondrial OXPHOS pathway. Instead of targeting aerobic glycolysis alone, a more efficient anticancer strategy is to target mitochondrial metabolism as well [104].

Arsenic trioxide (As_2_O_3_) is a classical anticancer drug used in the treatment of different forms of cancer. It can generate ROS and interfere with multiple signaling pathways [105]. A combination of As_2_O_3_ and dichloroacetate (DCA) inhibited BC cell proliferation, in which As_2_O_3_ disrupted mitochondrial function by targeting complex IV and DCA blocked PDK activity (Figure 1A, Table 1). Apart from targeting GLUT, HK, and LDHA, metformin can also inhibit complex I of the respiratory chain and also decrease citrate in TCA cycle [67].

## 4. Altered Glucose Metabolism and Anticancer Drug Resistance

Tumor metabolism does not only contribute to cancer progression but also to therapy resistance and can influence drug sensitivity. The increased demand for energy in resistant cells was consistent with the enhanced activity of drug efflux and drug detoxification mechanism of the cells [9]. The contribution of glycolytic enzymes in drug resistance is discussed in detail in Figure 1B.

### 4.1. Hexokinase and Drug Resistance

**Figure 2:**
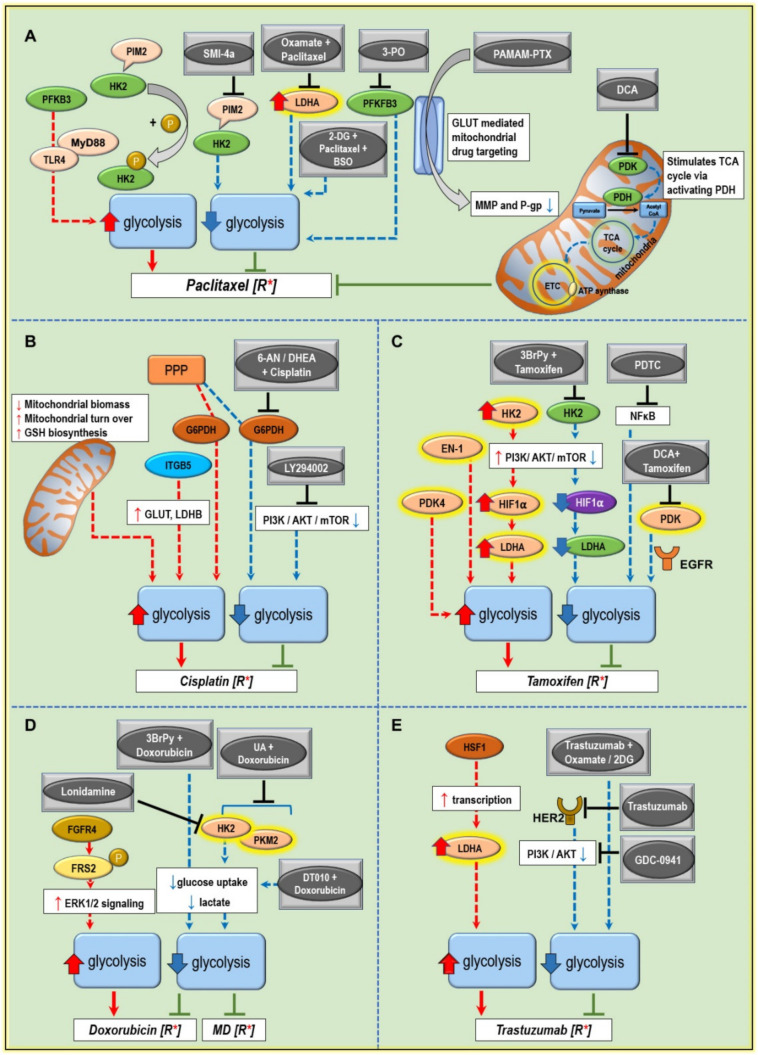
Illustrating the metabolic components contributing to drug resistance and drugs to overcome the resistance. The five panels show various signaling pathways converging to increased glycolysis and drugs as monotherapy or combinations that modify these pathways, which help to reverse the resistance mechanism. (**A**) Metabolic components contributing to paclitaxel resistance include upregulated expression of GLUT, HK2, LDHA, and PFKFB3, thereby switching the metabolism of cancer cells to aerobic glycolysis. Drugs targeting these enzymes potentially downregulate aerobic glycolysis, lactate production, and drug efflux pumps. PAMAM-PTX is a conjugated drug targeted to mitochondria via GLUT [115]. (**B**) Targeting PI3K/AKT pathway and G6PDH can reverse cisplatin resistance [116]. (**C**) Increased expression of EN, PDK4, HK2, and LDHA confers resistance to tamoxifen. PI3K/AKT pathway plays an essential role in Warburg’s effect [117]. (**D**) Various combinations of drugs can reverse the glycolytic phenotype and reverse multidrug resistance. Lonidamine and 3-BrPy are already in the clinical trial phase. (**E**) A combination of drugs targeting HER2 can downregulate PI3K/AKT and decrease aerobic glycolysis by resensitization of cancer cells to trastuzumab. Drug interventions that inhibit the non-glycolytic and tumor-promoting functions of the enzymes/pathway components are shown as black T-ended stop bar. The red arrows indicate components and pathways that contribute to drug resistance, thereby promoting tumor progression. In contrast, the blue arrows indicate components and pathways that contribute to the reversal of drug resistance/resensitization to the drug, thus supporting tumor suppression/regression. The green T-ended stop bar indicates the reversal of resistance/resensitization to the drug.

HK2 is highly expressed in cancers of the stomach, ovary, and cervix [106,107], and is associated with elevated glycolytic flux, which is a characteristic of cancer cells. Ahn K.J. et al. [88] investigated the role of increased expression and activity of HK2 in the proliferation of cancer cells. In hepatocellular cancer, they showed a twofold increase in cell survival and an eight-fold increase in resistance to cisplatin. Here, the non-glycolytic role of HK2 was mediated via PI3K/AKT/AMPK signaling. An in-depth study of HK2 function in ovarian cancer cell line revealed its extended role in migration, stemness, and invasion via FAK/ERK1/2/NANOG/SOX9 pathway [107]. A novel interaction between proviral insertion in murine lymphomas 2 (PIM2) and HK2 was discovered by Yang T. et al. [108]. PIM2 regulates aerobic glycolysis by phosphorylating HK2 (Figure 2A). Both PIM2 and HK2 are upregulated in BC tissue and contribute to paclitaxel resistance (Figure 1B) [108]. PIM2-mediated HK2 activity is a potential target for the treatment of BC [108]. Studies related to PIM2 showed that it regulates cell cycle progression, negatively regulates apoptosis, and correlates with malignant phenotypes. Thus, blocking PIM2 can induce apoptosis, cell cycle arrest, and senescence [108]. Studies report the role of HIF-1α in HK2 expression, e.g., inhibition of HK2 resensitizes tamoxifen-resistant cells (Figure 1B) [35] and HIF-1α, a transcription factor associated with hypoxia, is linked to cancer initiation.

### 4.2. PFKFB3 and Drug Resistance

6-Phosphofructo-2-kinase/fructose-2,6-bisphosphatase 3 (PFKFB3), an essential regulator of glycolysis, has the highest kinase activity among its four known isoforms and mediates interconversion of fructose-6-phosphate to fructose-2,6 bisphosphate. PFKFB3 has a critical influence on various stages of cancer progression, such as proliferation, drug resistance, angiogenesis, etc. [57]. Abnormal expression of PFKFB3 was observed in cancers of the brain, colon, breast, and ovaries [109]. Immunohistochemical analysis of 74 BC tissues revealed a positive correlation between PFKFB3 expression and metastasis and poor prognosis [94]. Additionally, it was demonstrated in MDA-MB-231 and MDA-MB-468 (TNBC) cells that the silencing of PFKFB3 inhibited proliferation, migration, and invasion and caused cell cycle arrest. Furthermore, PFKFB3 plays a vital role in angiogenesis and regulates the expression of p27, pAKT, and vascular endothelial growth factor alpha (VEGFα). HER2+ BC cell lines (SKBR3 and BT-474) showed elevated levels of PFKFB3 transcripts, which clinically correlated with poor prognosis [94]. A hypothesis based on the role of HER2 signaling in PFKFB3 expression and its contribution to BC progression was reported by O’Neal J. et al. [56], who showed that enhanced HER2 expression elevated glycolysis by increasing the activity of PFKFB3. Treatment with PFKFB3 inhibitor 3PO showed a reduction in tumor size in HER2+ mouse model of BC [56]. Klarer A.C. et al. [110] reported an interesting finding as they described the induction of autophagy as a prosurvival mechanism with 3PO treatment and thus recommended the use of an autophagy inhibitor along with 3PO for improved antitumor efficacy. Furthermore, inhibition of HER2 by lapatinib showed a reduction in glucose uptake and PFKFB3 transcript [56]. This proves that HER2 modulates tumor glycolysis by regulating PFKFB3 expression. A combination of PFKFB3 inhibitor and trastuzumab targeting HER2+ BC should be effective in resistant BC as well. PFK158, another PFKFB3 inhibitor, was shown to synergize with carboplatin and paclitaxel in the treatment of chemoresistant gynecologic cancers [111].

### 4.3. Enolase and Drug Resistance

Enolase (EN), a key glycolytic enzyme contributing to the Warburg effect, is used as a prognostic marker in lung cancer [112]. Moreover, EN is also an important marker in BC, as was demonstrated by an analysis of 244 BC tissue samples that revealed highly detected EN-1 in ER+ BC. Patients with high EN-1 showed poor prognosis with more extensive tumor volume, poor nodal status, and longer distance relapse [113]. Nevertheless, the knockdown of EN expression using siRNA significantly enhanced cytotoxicity to 100 nM tamoxifen in the treatment of tamoxifen-resistant BC cells. An upregulated EN-1 suppresses c-Myc expression, which results in the survival of resistant cells (Figure 1B) [36].

Additionally, proteomics profiling revealed that the silencing of EN-1 restored the oxidative phosphorylation by driving pyruvate to acetyl-CoA and TCA cycle in BC MDA-MB-231 cells. Apart from that, EN-1 silencing induced oxidative stress, senescence, and increased reactive oxygen species (ROS) production, followed by reduced glutathione in the same cell line [114]. Above all, targeting EN-1 is a novel strategy for treating tamoxifen-resistant BC (Figure 1A). EN-1 is also a right candidate as a prognostic marker for BC patients undergoing tamoxifen treatment [36].

### 4.4. Pyruvate Kinase and Drug Resistance

In the glycolytic pathway, pyruvate kinase (PK) catalyzes the conversion of phosphoenolpyruvate to pyruvate and generates ATP by transferring the phosphate moiety to ADP. In humans, PK exists in four isoforms: PKM1, PKM2, PKR, and PKL [118]. Apart from regulating glucose metabolism, research over the years has identified noncanonical functions of PK, which includes the modulation of gene expression, epigenetics, cell cycle regulation, and cell–cell communication [119]. An extensive review of PK by Israelsen W.J. and Van der Heiden M.G. [118] discusses multiple roles of PK and its function in the modulation of tumor microenvironment in cancer. Moreover, an interesting study by Hsu M.C. and Hung W.C. [119] mentions the prominent role of PKM2 in cancers and the metabolic switching from PKM1 to PKM2 isoform during carcinogenesis [120]. Additionally, a clinical study revealed a strong correlation between PKM2 and poor overall survival in cancers of the digestive system, but this was not confirmed in pancreatic cancer in which strong PKM2 expression correlated with improved overall survival [121]. Nevertheless, its role as a diagnostic marker in lung and colon cancer is contradicted by its poor specificity [122].

In BC, the association of PKM2 with VEGF-C was investigated in 218 BC patients in whom the mRNA levels of both were upregulated in tumor tissue compared to regular counterparts [123]. Moreover, the knockdown of PKM2 inhibited glycolysis and protein levels of VEGF-C, which subsequently reduced cancer cell proliferation in BC MCF-7 and MDA-MB-231 cells [124]. Combined expression of the above proteins in the clinical samples was associated with worse histological grade, lymph node metastasis, lymphovascular invasion, and poor patient survival [123,125]. Besides, the analysis of PKM1 and PKM2 expression in TNBC showed high expression of both proteins and targeting PK inhibited the proliferation of TNBC MDA-MB-231 and MDA-MB-436 cells via inhibiting NFκB signaling pathway [124]. Clinical studies reported a correlation between PKM2 expression and the prediction of chemosensitivity to epirubicin and 5-flurouracil (5-FU) based on the immunohistochemical analysis done in 296 patients diagnosed with invasive BC [126]. Other studies confirmed the inhibition of proliferation of cancer cells by decreasing the activity of PKM2. For example, combining paclitaxel with shikonin, a natural product isolated from *Lithospermum erythrorhizon* improved the efficacy to sensitize aggressive BC cells to paclitaxel [61]. Furthermore, inhibition of PKM2 using miRNA-122 overexpression resensitized resistant colon cancer to 5-FU [127]. In advanced BC, PKM2 expression correlated with cisplatin resistance [128]. Moreover, PKM2 enhanced chemotherapy resistance in ER+ BC models using MCF-7 and T47D cells through the promotion of aerobic glycolysis [129]. Conversely, a decreased PKM2 level was linked to cisplatin resistance in gastric carcinoma [130].

Overall, the significance of PKM2 as a prognostic marker depends on the type of cancer and the used chemotherapeutic agent. As mentioned before, a combination of markers could predict a more accurate clinical outcome in BC treatment.

### 4.5. LDHA and Drug Resistance

LDH is a key glycolytic enzyme in the conversion of pyruvate to lactate. LDHA is aberrantly expressed in many cancers, including breast, kidney, lung, and ovarian cancers [96,131,132]. Cancers relying on aerobic glycolysis generate more lactate [11]. ATP generated from aerobic glycolysis is predominantly utilized for tumor growth and metastasis. However, the knockdown of LDHA attenuated aerobic glycolysis and lactate production in murine 4T1 breast tumor cells [133]. The biochemical characterization of LDHA showed that phosphorylation at Y10 (tyrosine) confers metastatic potential in both in vitro and in vivo BC model. LDHA phosphorylation is regulated by HER2, whose expression is higher in BC tissue compared to healthy breast tissue [134]. LDHA phosphorylation at Y10 is a potential prognostic marker for metastatic BC. LDHA does not only mediate cancer progression, but it can also influence the sensitivity of BC cells to anticancer drugs. Studies investigating the role of LDHA in drug resistance reported a link between LDHA and paclitaxel resistance (Figure 1B) [62]. Oxamate, an inhibitor of LDHA, combined with paclitaxel-induced apoptosis in paclitaxel-resistant BC (MDA-MB-435 and MDA-MB-231) cells by inhibiting cellular glycolysis (Figure 2A). Therefore, LDHA is a potential therapeutic target for overcoming paclitaxel resistance and resensitizing BC to paclitaxel [62]. Moreover, the inhibition of LDHA also reverted the tamoxifen-resistant phenotype by inducing apoptosis and inhibiting the prosurvival autophagy in tamoxifen-resistant BC (MCF-7 and T47D) cells [135]. Independent studies showed a relatively higher expression of LDHA and AMPK activation in TNBC cells [96]. Analysis of TNBC tissue samples demonstrated a stronger correlation of LDHA and AMPK with distant metastasis, Ki67, and overall survival [96,136]. Interestingly, the LDHB isoform was differently expressed within various subtypes of TNBC and predicted a basal-like subtype of TNBC. LDHB isoform was reported low in hormone receptor-positive/HER2-negative cancers [137].

### 4.6. PDH/PDK and Drug Resistance

Pyruvate dehydrogenase (PDH) is a part of the pyruvate dehydrogenase complex (PDC) in the glycolytic pathway converting pyruvate to acetyl-CoA [138]. PDH is regulated by the inhibitory action of pyruvate dehydrogenase kinase and is reactivated by pyruvate dehydrogenase phosphatase depending upon changes in the levels of pyruvate/acetyl CoA and NADH levels [139]. Under pathological conditions like cancer, this regulation is substantially altered [140]. An upregulated PDK is implicated in many cancers; its role in aerobic glycolysis, drug resistance, and metastasis has been well documented [141]. Furthermore, studies reported enhanced expression of PDK1 isoform promoting BC stem cell (BCSC) properties [142]. Another group reported the involvement of PDK4 isoform in regulating aerobic glycolysis and mitochondrial respiration in BC. Its high expression correlated with poor prognosis in BC treatment irrespective of BC molecular subtypes.

Further investigation revealed the transcriptional regulation of PDK4 by miRNA-211, while inhibition of PDK4 by miRNA-211 induced a shift from glycolytic phenotype to OXPHOS dominant phenotype in BC MDA-MB-468 and BT-474 cells [138]. Furthermore, Walter W. et al. [143] identified the altered regulation of PDH by PDK4 and the association of PDK4 and anti-estrogen resistance in BC cells [143]. However, in colorectal cancer tissue, PDK1 had no role, but its PDK3 isoform was associated with cancer progression. Moreover, hypoxia-mediated enhanced expression of PDK3 inhibited drug-induced apoptosis, thus conferring drug resistance in colorectal cancer cell lines (HCT116 and HT29) [144]. Recent research suggests that inhibition of PDK can control cancer growth by regulating aerobic glycolysis [145]. Aspirin could effectively reduce the stemness and aerobic glycolysis in BC cell lines by inhibiting PDK1 and stemness related factors octamer-binding transcription factor 4 (OCT4), sex-determining region Y-box 2 (SOX2), and homeobox transcription factor (NANOG) [142]. Combining PDK1 inhibitors (triciribine and tetrandrine) with tamoxifen was found to resensitize BC to tamoxifen treatment [146]. Therefore, PDK inhibitors are potent anticancer agents [147]. Furthermore, new studies highlight the critical role of mitochondrial pyruvate carrier (MCP) in tumor energy metabolism. In a recent study, inhibition of MCP by 7ACC2 resulted in decreased lactate uptake by cancer cells for its energy requirements. In vivo studies showed that MCP inhibition reduced tumor volume and increased radiosensitization of tumors [148].

## 5. Modulating Glucose Metabolism to Increase the Anticancer Drug Efficiency and Combat Treatment Resistance

A growing body of literature asserts the role of tumor metabolism in the transformation process in cancer. Drugs modifying glucose metabolism positively favor the action of conventional chemotherapeutic drugs commonly used for cancer treatment. The following section discusses the metabolic alterations in the glucose metabolism in cancers associated with resistance towards conventional drugs used in BC and the possible ways to resensitize aggressive BC phenotypes (Figure 2).

### 5.1. Paclitaxel Resistance

Paclitaxel is a chemotherapeutic drug frequently used in TNBC treatment [149]. Though the initial response to paclitaxel is impressive, the majority of BC patients often develop resistance, ultimately leading to relapse, metastasis, and death [150].

A comparative in vitro study in a paclitaxel-resistant and -sensitive BC (MCF-7) cell line showed higher resistance to paclitaxel, cross-resistance to doxorubicin, and prolonged doubling time. Resistant cell lines also showed low ER status and upregulated HER2 and Ki-67 expression. Other resistant factors found in the multidrug-resistant (MDR) phenotype, such as P-glycoprotein (P-gp), lung resistance-related protein (LRP), and glutathione-S-transferase-π (GST-π), were also upregulated in the resistant cell line [151]. The underlying mechanisms contributing to the paclitaxel resistance are multiple. The most common mechanism seen in paclitaxel resistance is drug efflux by ABC transporters [152] and the involvement of drug detoxification enzymes cytochrome P450, CYP3A4/5, and CYP2C8 [153]. The energy required for drug efflux is derived mainly from the glycolytic pathway [154]. An interesting study conducted by Zuo K.Q. et al. [155] compared paclitaxel binding proteins between resistant MCF/Paclitaxel cell line and the sensitive phenotype, and they identified proteins such as heat shock protein 90, actinin and dermcidin precursor being absent in the resistant phenotype. The dermcidin precursor is associated with metabolism and has a prominent role in tumor cell survival [156]. As previously reported, paclitaxel induces oxidative stress in BC MDA-MB-231 and T-47D cells [157].

Nevertheless, when combined with 2-DG (glycolytic inhibitor) and buthionine sulfoximine (BSO) a glutathione synthesis inhibitor, the sensitivity towards paclitaxel was enhanced due to hydrogen peroxide (H_2_O_2_) and superoxide (O_2_^•−^) production (Figure 2A). 2-DG was tested at pre-clinical or phase I/II clinical trial for BC treatment [158]. As mentioned in Section 4.5, LDHA, which is predominantly expressed in BC tissue, has a significant role in nutrient exchange within the tumor microenvironment [62,159]. In vitro studies in the paclitaxel-resistant BC MDA-MB-435 cell line showed a 2-fold increase in LDH activity. The half maximal inhibitory concentration (IC_50_) values from cell viability assay were 30-fold higher compared to the parental cell line. LDHA expression and its activity play a direct role in the paclitaxel resistance. Downregulation of LDHA using siRNA increased the sensitivity to paclitaxel by 2-fold, showing that targeting glycolytic enzyme can resensitize the resistant cell to paclitaxel. Consistent with the above observations, siRNA mediated knockdown of LDHA and combining paclitaxel with oxamate, an analog of pyruvate, significantly inhibited the viability and induced apoptosis in paclitaxel-resistant BC cells (Figure 2A) [62]. Likewise, in vivo studies underlined that paclitaxel and oxamate combination induced synergistic cytotoxic effects, apoptosis, and antiangiogenic activity in a murine BC model [160].

Moreover, PFKFB3, a critical glycolytic regulator, was examined for its role in paclitaxel resistance in hormone receptor-positive BC (MCF-7RA and MCF-7RB) cell lines. The silencing of PFKFB3 markedly reduced the IC_50_ concentrations in resistant phenotypes of both BC cell lines. Furthermore, upon paclitaxel exposure, PFKFB3 regulated the expression of TLR4, MyD88, and interleukin release in the tested cell lines (Figure 2A) [161].

Dysregulated cell cycle machinery and the subsequent uncontrolled proliferation of cells are commonly featured in all cancers. Cell cycle components cyclin D and E, CDK4/6, and CDK2 show altered expression in TNBC [162]. Cell cycle inhibitors are commonly used in the treatment of ER+, HER2 − BC patients [163]. However, the cell cycle inhibitor against CDK4/6 has proven efficiency in a subtype of TNBC whose genotype is Rb-positive, p16^INK4^-negative [162]. Recently, a study tested the efficacy of CDK4/6 inhibitor, palbociclib, along with paclitaxel in TNBC MDA-MB-231 and HCC38 cell lines following a different time and exposure regime (simultaneous or sequential). The simultaneous application of CDK4/6 inhibitor along with paclitaxel encountered an antagonistic effect in both cell lines.

Conversely, the sequential application of palbociclib followed by exposure to paclitaxel inhibited proliferation and induced apoptosis of tested cells. Mechanistically, pretreatment of cells with palbociclib and the subsequent removal of palbociclib before paclitaxel application helped to synchronize the cell cycle re-entry from the G1 phase to S phase, which rendered cells susceptible to the cytotoxic drug. Above all, palbociclib is capable of reprograming the glucose metabolism by downregulating GLUT-1 glucose uptake via Rb/E2F/c-Myc signaling pathway. It also inhibits HIF-1α expression, a key regulator of tumor progression [162].

Another feature observed in the paclitaxel-resistant cells is the decreased level of citrate and defective mitochondrial respiration. As observed in human lung adenocarcinoma A549 cells, dichloroacetate (DCA) can normalize mitochondrial function by inhibiting pyruvate dehydrogenase kinase (PDK) and reverse paclitaxel resistance. Inhibition of PDK activates pyruvate dehydrogenase complex (PDC), thereby activating the conversion of pyruvate to acetyl CoA and entry of the later into TCA cycle to form citrate. This process activates the oxidative phosphorylation in mitochondria. Additionally, DCA inhibits the activity of P-gp protein associated with drug efflux by inhibiting glycolysis [164] (Table 2).

Moreover, the aberrant Wnt signaling pathway is highlighted in many cancers, including BC, and is associated with poor prognosis [165]. A review of Wnt signaling highlights its role in tumor metabolism [166]. A dysregulated canonical Wnt/β-catenin pathway through c-Myc enhances proliferation, aerobic glycolysis, alanine-serine-cysteine transporter 2 (ASCT2) levels, and glutathione levels contributing to therapy resistance [166]. A comparative study between resistant and parent (MCF-7) cells showed increased expression of HER2 and β-catenin in the resistant phenotype. The neuroleptic agent penfluridol suppressed chemoresistance of paclitaxel-resistant BC cells by modulating HER2/β-catenin signaling via downregulating HER2, β-catenin, c-Myc, and cyclin D1 [167]. Targeted therapies towards Wnt signaling are currently evaluated in the pre-clinical/clinical trial stage [165]. Furthermore, ursolic acid (UA), a plant-derived triterpene, reversed the chemoresistance to paclitaxel in BC MDA-MB-231 cells by targeting MyD88 via miRNA-149-5p. The miRNA-149 plays a significant role in suppressing different oncogenes and is frequently downregulated in BC. Overexpression studies with miRNA-149 demonstrated an inhibition of PI3K/AKT signaling pathway and MyD88 and a reverse of resistant to sensitive phenotype [168]. Additionally, an alkylating agent temozolomide exerted its anticancer effect by downregulating glucose metabolism. A combination of temozolomide and paclitaxel can synergistically inhibit paclitaxel-resistant brain tumors (gliomas) [169].

### 5.2. Cisplatin Resistance

Cisplatin (cis-diamminedichloroplatinum (II)) is a cytotoxic drug commonly used in the treatment of cancers of the breast, cervix, head, neck, and prostate [176,177]. Its mechanism of cytotoxicity is mainly associated with the formation of DNA adducts, eventually inducing apoptosis in cancer cells. Though cisplatin is a potent cytotoxic agent, the emergence of resistance is often seen during long-term treatment in which the tumor fails to respond to clinically relevant dosages. Resistant tumors respond only to almost double the standard concentration. Factors contributing to cisplatin resistance are many and include reduced drug intake, enhanced drug efflux, and drug detoxification. Resistance to cisplatin is also associated with cell signaling components such as PI3K/AKT and HER2 expression. Additionally, an attenuation of Ras and MAPK pathway also mediates cisplatin resistance [178].

The glycolytic components conferring resistance to cisplatin include altered glycolytic enzymes and glucose transporters [179]. Better observation of the metabolic role in cisplatin resistance was described in the ovarian cancer cell line [116]. Compared to cisplatin-sensitive ovarian cancer cells, the resistant cells showed no change in glucose uptake at an early point of drug application. Interestingly, protein levels of GLUT-1 showed no change, but a change was observed in the localization of GLUT-1 after cisplatin treatment of tested cells. It is possible that cisplatin can bind to tubulin and can inhibit the transportation of GLUT-1 to the plasma membrane [180]. Another study reported a downregulation of GLUT-1, GLUT-4, and LDHB via integrin β5/focal adhesion kinase (ITGB5/FAK) signaling in MDA-MB-231 (TNBC) and cervical cancer cell line Siha (Figure 2B) [63]. Besides, a fusion of cisplatin and dichloroacetate-platinum (II)-DCA reported an efficient cytotoxic effect in ovarian cancer cell line by a mechanism based on the dual targeting of DNA and mitochondria [181].

Similarly, based on the principle of dual targeting, a novel mitochondrial inhibitor, 4-(N-(S-penicillaminylacetyl) amino) phenylarsonous acid (PENAO), along with DCA demonstrated cytotoxicity in BC T47D and MDA-MB-231 cells [182]. Despite that DCA was also tested in a clinical trial, an evaluation of its response rate in patients with metastatic BC was not associated with clear results [170]. Moreover, a combination of metformin and cisplatin had a synergistic effect on cisplatin-resistant TNBC (Hs 578T and MDA-MB-231) cells by a mechanism not directly related to the glycolytic pathway. Here, metformin downregulated the expression of RAD51, a protein often linked to resistance to DNA damaging cytotoxic agents. However, the antineoplastic efficiency of metformin was reduced by high external glucose concentration [183].

### 5.3. Tamoxifen Resistance

Tamoxifen is administered in patients with ER+ BC [184]. BC, which initially responds to tamoxifen, often develops resistance to treatment [185]. Tamoxifen is a selective ER modulator [186]. ER plays a vital role in breast tumorigenesis by activating the survival pathway and can influence glycolysis by the regulation of GLUT-1 expression. Tamoxifen can inhibit mitochondrial respiratory complex I, thereby decreases ATP level and subsequently activates the AMPK signaling, leading to the induction of apoptosis through mTOR inhibition. Irrespective of ER status, tamoxifen affects tumor metabolism [117,187]. This indicates a mechanism independent of classical modulation of ER. A review by Manna S. and Holz M.K. [188] mentions that 5–10% of ER-ve BC responds to Tamoxifen treatment. The mechanism involves ER-independent signaling components such as AKT phosphorylation, estrogen-related receptor alpha (ERRα), TGFβ signaling, and characteristics of the tumor microenvironment. Tamoxifen demonstrated metabolic restabilization in BC (MCF-7 and MDA-MB-231) cells when combined with glycolytic inhibitor 2-DG or 3 bromopyruvate (3-BrPy) and in vivo data also showed similar results (Table 2) [189]. Another study demonstrated a two-fold increase in GLUT-1 expression in MCF-7 cells when stimulated with estrogen, while the presence of tamoxifen decreased this effect [44]. Besides, enhanced expression of HK2 and mTOR was reported in the tamoxifen-resistant MCF-7 cell line. Mechanism of tamoxifen resistance showed enhanced autophagy by HK2 via inhibition of the mTOR-S6K signaling [35]. A lower mTOR activity conferred the resistance to the drug. In tumors, both aerobic glycolysis and HIF-1α stabilization can occur even in the absence of hypoxia driven by oncogenic signals. Woo Y.M. et al. [117] reported that AKT/mTOR pathway or AMPK signaling could activate HIF-1α even in the absence of hypoxia [117]. Inhibition of HK2 suppressed AKT/mTOR/HIF-1α axis and resensitized tamoxifen-resistant BC cell lines derived from MCF-7. Treatment with 3-BrPy, an inhibitor of HK2, reduced the cell viability as indicated by decreased glycolytic rate and lactate accumulation as well as cell cycle arrest at G1 phase (Figure 2C) [117]. Another key glycolytic enzyme, EN-1, also plays a role in tamoxifen resistance [36]. Enhanced EN-1 expression promotes cell survival by negative regulation of c-Myc promoter-binding protein (MBP-1)/c-Myc gene. Pretreatment of the tamoxifen-resistant MCF-7 cells with pyrrolidine dithiocarbamate (PDTC), an inhibitor of NFκB, resensitized them to tamoxifen by inhibiting transcriptional regulation of EN-1 by NFκB, thereby lifting inhibition of c-Myc by NFκB [36] (Figure 2C). A combination of tamoxifen and DCA inhibited the growth of tamoxifen-resistant MCF-7 cells by downregulating EGFR signaling [187]. All studies identify a common mechanism, specifically the involvement of AKT/mTOR/AMPK signaling in tamoxifen resistance in BC [117,190,191].

### 5.4. Doxorubicin Resistance

Doxorubicin (Adriamycin) is a drug widely used both in the early and late stages of BC [192]. Toxic side effects and the emergence of resistance is a common feature associated with doxorubicin treatment [192]. Multiple factors can contribute to doxorubicin resistance. Microarray analysis of MDA-MB-231 cells identified some critical proteins associated with doxorubicin resistance, which included cyclin D2, cytokeratin 18, and heterogeneous nuclear ribonucleoprotein m3-m4 [193]. P-gp, a significant factor contributing to drug efflux, is an essential player in drug resistance [194]. P-gp belongs to ATP-binding cassette family and requires energy in the form of ATP for functioning [195]. Some studies link glycolysis with drug efflux transporters [196]. Effects of 3-BrPy, a glycolytic inhibitor, was tested in doxorubicin-resistant neuroblastoma (NB) cells. 3-BrPy with doxorubicin synergistically inhibited cell viability of NB cells under both normoxic and hypoxic conditions. Consistent with the cell viability, total ATP levels, and lactate levels were reduced upon combination treatment with 3-BrPy with doxorubicin [196]. Here, the author assumes that 3-BrPy reduces the intracellular ATP levels, thereby affecting drug efflux transporter function, which is ATP-dependent and helps in anticancer drug retention within the cancer cell.

Moreover, the sensitivity to doxorubicin was compared in 2D and 3D cell culture models (MCF-7 and MDA-MB-231) of BC. This study identified a distinct factor, cell-to-ECM interactions, contributing to doxorubicin resistance in BC. Integrins play an essential role in cell-to-ECM interactions; therefore, inhibition of β1-integrin using antibody before doxorubicin treatment enhanced its cytotoxic effect [192]. Integrins have multiple functions, which include adhesion, migration, and proliferation, that are controlled by signaling pathways such as AMPK, mTOR, and HIF-1 (the role has been mentioned under tamoxifen resistance), and in turn, they also control other glucose metabolic pathway via β1-integrin/FAK/PI3K/AKT/mTOR signaling [197]. A combination of DT-010, a conjugate of danshensu (DSS) and tetramethylpyrazine (TMP), and doxorubicin synergistically inhibited the viability of MCF-7 cells by downregulating glycolysis and inhibiting GRP78, a regulator of ER stress and aerobic glycolysis [198]. Another combination of doxorubicin with ursolic acid (UA) in doxorubicin-resistant MCF-7 cells, resulted in improved intracellular accumulation of doxorubicin probably by the inhibition of P-gp mediated by UA (Figure 2D) [199].

Moreover, metabolic alteration in doxorubicin-resistant BC cells revealed enhanced Fibroblast Growth Factor (FGF)–Fibroblast Growth Factor Receptor (FGFR) signaling and the activation of downstream signaling controlling various oncogenic processes such as angiogenesis, therapy resistance, and metastasis. Gene expression microarray revealed the role of FGFR in increased glycolytic flux and chemoresistance to doxorubicin. Blocking of FGF-FGFR-ERK1/2 signaling by pharmacological inhibitors targeting FGFR4 and ERK1/2 (PD173074 or U0126) resensitized resistant phenotype to doxorubicin treatment [200] (Figure 2D). Metformin, an antidiabetic drug, has demonstrated its antiproliferative effects in various BC cell lines and was able to sensitize the MDR phenotype [201]. A combination of metformin (6 µM) and doxorubicin in the resistant phenotype of MCF-7 and MDA-MB-231 cells presented more cytotoxicity than doxorubicin alone, while metformin acted via IFN-α signaling pathway and by induction of cellular oxidative stress [202].

### 5.5. Trastuzumab Resistance

A recent review by Hoxhaj G. and Manning B.D. [203] highlights the significant role of PI3K/AKT pathway in the oncogenic process, including tumor energy metabolism. PI3K/AKT signaling has multiple control points in the glucose metabolic pathway, including glucose transporters and enzymes regulating glycolysis.

Trastuzumab (Herceptin), an antibody directed against HER2, is the first targeted therapy approved for HER2+ BC patients [204]. Mechanistically, trastuzumab inhibits the HER2 (ErbB2) receptor, and downstream signaling reduces the PI3K/AKT signaling by restoring PTEN function. Thus, trastuzumab, along with PI3K/AKT inhibitors, potentially increases the efficacy of cancer treatment [205]. A study of the molecular mechanism of trastuzumab action demonstrated inhibition of aerobic glycolysis via ErbB2-heat shock factor 1 (HSF1)-LDHA pathway (Figure 2E) while the sensitivity to trastuzumab was associated with LDHA activity [124]. HSF1 transcriptionally regulates LDHA, and a knockdown of HSF1 was associated with repressed LDHA expression in BC BT474 and SKBR3 cells (Figure 2E). Overexpression of HSF1 decreased the sensitivity of tested cancer cells to trastuzumab, thus confirming the role of HSF1 in the resistance (Figure 2E). Acquired resistance to trastuzumab is frequently encountered in metastatic BC [204]. Another key glycolytic enzyme valuable for assessing trastuzumab response in advanced metastatic BC is PKM2, while its level in the plasma is used as a marker for monitoring therapeutic progress [206]. As cancer is associated with altered metabolic phenotype, combining anticancer agents with glycolytic inhibitors is a promising strategy for improving treatment efficiency [207]. Combinations of trastuzumab with either 2-DG or oxamate synergistically inhibited cancer growth both in vitro and in vivo in comparison with these compounds alone [204].

### 5.6. Palbociclib Resistance

Palbociclib is one of the targeted therapies prescribed for (ER+/HER2−) BC patients undergoing endocrine therapy [208]. The development of resistance is observed in 50% of patients in later stages of treatment [136]. A study highlighted a difference in glucose metabolism among ER+/HER2−/+ subtypes of BC cell lines exposed to palbociclib. Enhanced aerobic glycolysis was observed in ER+/HER2− palbociclib sensitive cells, whereas increased glucose catabolism was observed in ER+/HER2+ palbociclib resistant cells [209]. Moreover, synergistic effects with palbociclib and endocrine therapy were reported in preclinical models [208].

## 6. The Scope of Targeting Warburg’s Effect for Resensitizing BC Chemotherapy

The adaptability of neoplasms to switch between conditions of normoxic and hypoxic microenvironment confers a high degree of metabolic heterogeneity in various cancers, which in turn supports proliferation, growth, and progression while evading apoptosis [210]. Depending on their energy requirements, cancer cells can switch between various signaling pathways for resourcing metabolites from other pathways (such as glutamine, PPP, and fatty acid metabolism). Under this situation, the metabolic landscape of the tumor varies from the core towards the periphery of the tumor [211]. Therefore, the metabolic map derived from a tumor tissue may not represent the tumor as a whole [210]. Therefore, targeting a transient metabolic phenotype may be confusing from a therapeutic point of view unless otherwise, the target represents a permanent metabolic perturbation.

Therapy resistance is frequently encountered in the course of BC treatment in both endocrine-based therapy (for hormone receptor-positive BC) and chemotherapy (for TNBCs). Evidence shows that tumor metabolism is linked to the development of drug resistance. Targeting enzymes regulating energy metabolism is an efficient strategy complementing conventional treatment of BC. These targets are useful diagnostic and prognostic markers. Biomarkers representing the metabolic phenotypes are more useful prognostic markers, as in the case of HK2 and LDHA, which represents overall patient survival, metastasis, and progression [37,96].

Interestingly, EN-1 is specifically related to resistance in ER+ BC. Therefore, targeting EN-1 could be beneficial in tamoxifen-resistant BC. Glycolytic inhibitors, like lonidamine, 3-BrPy, and 2-DG, in combination with anticancer agents, have encouraging results in pre-clinical studies. Reports also suggest the existence of a small group of breast cancer stem cells (BCSCs) that support tumor sustenance and have a critical role in the resistance to chemotherapy and radiotherapy. The proteomic and targeted metabolomic analysis showed metabolic alterations and adaptive mechanisms in BCSCs, such as a very high level of HIF-1α that is predominantly relying on aerobic glycolysis [212].

Moreover, tumor-associated macrophages promote aerobic glycolysis and resistance to apoptosis of BC cells through extracellular vesicle transmission of HIF-1α stabilizing long noncoding RNA (HISLA), while its blockage inhibits glycolysis and chemoresistance of BC in vivo [213]. Literature reports that glycolytic enzymes, including HK, PKM2, LDHA, and PDK, are related to BC stemness [212]. Therefore, inhibitors of these dysregulated enzymes are possible solutions to resistance in BC. 2-DG alone or along with doxorubicin combination overcomes drug resistance by eliminating BCSC [212].

### 6.1. PI3K/AKT Pathway is a Crucial Signal Contributing to Therapy Resistance

In the 1920s, Warburg first reported a noticeable difference in the bioenergetics of cancer compared to healthy cells showing increased glucose metabolism and lactate production in tumor cells [10]. Many authors later confirmed this hypothesis by demonstrating the overexpression and dysregulated activity of key glycolytic enzymes (HK, PFKFB3, PK, and LDHA) and transporters (GLUT and MCT), followed by altered non-canonical function (here non-metabolic functions) of these enzymes [214]. HIF-1α, as a significant contributor to the Warburg effect, is associated with the regulation of many metabolic gene expressions [215]. Aberrant expression of these enzymes can facilitate oncogenic signaling, such as continuous proliferation signaling, migration, and inhibition of apoptosis [215]. Many signaling pathways are abnormally activated in drug resistance. The most crucial signaling regulating tumor metabolism is PI3K/AKT/mTOR [216]. This pathway, in turn, enhances the activity of HK, PK, and LDH enzymes of the glycolytic pathway and increases the glucose uptake in cancer cells and promotes the emergence of resistance [13]. Based on the literature reviewed in this current article, in the context of tumor metabolism, acquired resistance to chemotherapy is predominantly regulated by PI3K/AKT signaling [14,216,217]. PI3K/AKT confers resistance via multiple pathways such as inhibiting apoptosis through the downregulation of p53, activation of NFκB and subsequent transcriptional activation of drug efflux pumps, and survival signaling [218]. Therefore, multiple pieces of evidence support the fact that inhibition of PI3K and AKT could be useful in reversing the chemo-resistance of cancer cells.

### 6.2. Targeting Glycolytic Enzymes Prove to Be Beneficial In Both In Vitro and In Vivo Cancer Models

Downregulation of vital glycolytic enzymes can resensitize cancer to treatment by blocking aberrant metabolic pathways. Timing and sequence of administration of drugs can have two different outcomes, i.e., antagonistic or synergistic effect, as in the case of palbociclib/paclitaxel combination in which the simultaneous application of two drugs resulted in antagonistic effect whereas pretreatment for 24 h of palbociclib followed by paclitaxel showed an additive effect. These findings from the in vitro studies can be used in clinical settings when scheduling a treatment regime [162]. Paclitaxel resistance in lung adenocarcinoma cells is characterized by a defective mitochondrial respiratory function and decreased citric acid levels. Regulating glucose metabolism by activating TCA cycle can be an alternative strategy to reduce resistance, as in the case of paclitaxel treatment (Figure 2A) [164]. Moreover, insulin treatment caused a twofold increase in the cytotoxic effect of 5-FU and cyclophosphamide in BC MCF-7 cells [219]. Clinical studies also demonstrated the chemosensitization effect of insulin in BC patients undergoing chemotherapy. In a clinical trial involving multidrug resistant metastatic BC (resistant to fluorouracil + Adriamycin/doxorubicin + cyclophosphamide), a combination of insulin administration enhanced the antitumoral response to methotrexate. It significantly lowered the median increase in tumor size when compared to those who were administered with either insulin or methotrexate alone [220]. However, the mechanism of insulin-mediated potentiation of chemosensitivity is not clear yet. Understanding the underlying mechanism of drug resistance is essential to design improved drug molecules.

### 6.3. Novel Methods to Improve Anticancer Drug Efficacy by Modulating the Tumor Glucose Metabolism

Disruption of glucose metabolism for selective targeting of cancer is a novel approach, but the feasibility of targeting specific enzymes of the cancer tissue without affecting the healthy cells is not easy. A possible solution could be the novel PROteolysis Targeting Chimera (PROTACs) technology [221]. This technology uses ubiquitin-proteasome machinery to degrade undesirable cancer-promoting proteins. PROTACs is a bifunctional molecule with a ligand for recruiting the ubiquitin-proteasome and a second ligand to link to the target protein explicitly [179]. This method was successfully demonstrated in MCF-7 cells treated with HIF-1-estrogen Protac, in which the results showed functional downregulation of ER and subsequent growth inhibition [222]. However, this technology needs to be further advanced before being introduced to clinical trials. Based on the principle of Warburg’s effect, another novel method for specific targeting of a tumor is glyco-conjugation of drugs, which ensures selective delivery of the cytotoxic drug to cancer cells (e.g., glufosfamide and 2-DG-conjugated paclitaxel) [223,224]. This technique utilizes highly expressed GLUT transporters expressed in cancers for improved uptake into the cells, thus minimizing side effects. More advanced drug designs, such as multicomponent conjugate drugs, demonstrate high efficacy and selectivity in targeting cancer cells. For example, unlike poorly soluble paclitaxel, PAMAM-paclitaxel-trastuzumab is a potent target-based drug, with increased permeability for HER2+ (SKBR-3) BC cells [115].

### 6.4. The Power of Combinations for Enhanced Efficacy of Chemotherapy in BC

The principle behind combination therapy is to combine drugs of different mechanisms to improve the overall anticancer efficacy and reduce drug resistance. Combinatory therapy produces a synergistic or additive effect compared to monotherapies. The use of anti-glycolytic inhibitors is beneficial in cancers in hypoxic conditions or with mitochondrial defects. Metformin, an antidiabetic drug, clinically showed improved chemotherapeutic and radiotherapeutic activity in combination with anticancer agents [45]. The combination of a more potent version of metformin, called phenformin (50 times), and oxamate was reported to be effective in various cancer cells, including BC [225]. Clinical data supports the finding that combining the classical anticancer drug with metabolic inhibitors resensitizes cancer to conventional chemotherapy and radiotherapy [226]. The selection of combination drugs should be guided by the analysis of tumor metabolic biomarkers in patient samples. In the case of heterogeneous tumors like TNBC, understanding the molecular or metabolic landscape can help in the selection and prediction of drug response, especially for those markers that are noninvasive and quantifiable and may be beneficial for continuous evaluation of treatment [19,44,227].

## 7. Conclusions and Future Perspectives

This review provides a comprehensive analysis of mechanisms behind metabolic alterations conferring resistance to anticancer drugs and highlighting the reprogramming of tumor metabolism using metabolic inhibitors to sensitize cancer to cytotoxic drugs. A better understanding of metabolic characteristics and the molecular classification of BC helps to redefine the therapeutic approach. A primary challenge in targeting tumor metabolism for cancer treatment is changing the metabolic characteristics of a tumor during its progression and plasticity of cancer cell to switch between alternative pathways for energy, survival, and inhibition of apoptosis [228]. Additionally, in many cases, during targeting terminally differentiated cancerous cells, less abundant cancer stem cells are capable of evading the anticancer effect of the drug [229]. These cancer stem cells possess unique metabolic characteristics. They are capable of evading the immune response mechanisms and express essential transporter proteins that support multidrug resistance in addition to its ability to self-renew and differentiate into tumor progenitor cells, which then promotes cancer invasion, metastasis, and relapse [229]. Therefore, anticancer strategies must be efficient enough to target both terminally differentiated cancer cells and cancer stem cells [229]. Proteomic and metabolomic studies on tumor metabolism bring physicians closer to the successful identification of targets and hence a more effective clinical translation. Further research into tumor cell and cancer stem cell metabolism is warranted for overcoming the limitations in targeted therapy. The ongoing repurposing of drugs and the introduction of new candidate drugs that target tumor cell and cancer stem cell metabolism are promising research avenues and therefore deserve considerable attention.

## Figures and Tables

**Table 1:** A list of glycolytic inhibitors targeting critical enzymes in the glucose metabolic pathway.

Serial Number	Target	Compounds	Model	Reference
1	GLUT	Apigenin, Genistein, Cisplatin, Metformin, Tamoxifen, EGCG, Hesperetin, Kaempferol, Silybin	MCF-7, ZR-75-1, T-47D, MDA-MB-231, 4T1	[42,44,45,46,47,48,49,50,51]
2	MCT	Quercetin, α-cyano-4-hydroxycinnamate and Lonidamine	MDA-MB-231, MDA-MB-468, MCF-7/AZ, SKBr3, Hs 578T, BT-20	[52,53]
3	HK	Polydatin, 2-DG, Tamoxifen, Metformin, 3-BrPy, EGCG	MDA-MB-231, 4T1	[54,55]
4	PFKFB3	3PO, PFK15, PFK158	SKBR3, BT-474	[56,57]
5	GAPDH	3-BrPy	HepG2, PC-3	[58,59]
6	PK	Vit K3, Vit K5, Shikonin, 5-FU, Lapatinib, EGCG, Quercetin	MDA-MB-231 MCF-7, 4T1	[60,61]
7	LDHA LDHB	Cetuximab, Metformin, Oxamate, Chidamide, Galloflavin Cisplatin	MBA-MD-231 MCF-7	[46,62,63,64,65]
8	Mitochondria	DCA, Metformin, As_2_O_3_	T47D, BT-20, MCF-10A, MDA-MB-468, MDA-MB-231	[66,67,68,69]

2-DG = 2-deoxyglucose, 3-BrPy = 3-bromopyruvate, 3PO = 3-(3-pyridinyl)-1-(4-pyridinyl)-2-propen-1-one, 5-FU = 5-flurouracil, DCA = dichloroacetate, EGCG = epigallocatechin-3-gallate, GAPDH = glyceraldehyde-3-phosphate dehydrogenase, GLUT = glucose transporter, HK = hexokinase, LDHA/B = lactate dehydrogenase A/B, MCT = monocarboxylate transporter, PFKFB3 = 6-phosphofructo-2-kinase/fructose-2,6-bisphosphatase 3, PK = pyruvate kinase.

**Table 2:** Combination therapy with glycolytic inhibitors in BC chemotherapy.

Drug	Model	Dose	Observation	Side Effects	Mechanism	Study Method	Ref.
DCA + cisplatin	Stage IV BC (1 case)	DCA 6.25 mg/kg	–	Pulmonary embolism, edema	–	NCT01029925 (2014)	[170]
DCA	Rat mammary adenocarcinoma	200 mg/kg/day. (1.5–3mM plasma level)	50% reduction in lung metastasis	Minimal side effects	Inhibition of PDK	In vivo	[68]
Oxamate + paclitaxel	Solid Ehrlich Carcinoma	Oxamate (300 mg/kg) and paclitaxel (10 or 20 mg/kg)	>40% Reduction in volume of SEC, ATP, IL-17	–	LDH-A inhibition induced apoptosis	In vivo	[160]
Doxorubicin + metformin + oxamate	TNBC xenograft	Doxorubicin 1 mg/kg, Metformin 200 mg/kg, and Oxamate 15 mg/kg	Reduced tumor volume	–	LDH-A inhibition induced apoptosis	In vivo	[171]
Doxorubicin + lonidamine	Metastatic BC patients	Doxorubicin 75 mg/m^2^/21day + lonidamine 600 mg/m^2^/day	Overall response rate is 68% in BC with liver metastasis	Cardiotoxicity, myalgia	–	Randomized clinical trial (1998)	[172]
Tamoxifen + 3-BrPy	Solid Ehrlich Carcinoma	Tamoxifen 5 mg/kg and 3-BrPy 10 mg/kg	80% reduction in tumor volume, increased oxidative stress	–	Decreased MMP-2/9, VEGF	In vivo	[173]
Trastuzumab + metformin Paclitaxel + trastuzumab + metformin	Xenograft HER2+ primary BC	Trastuzumab 5 mg/kg/once a week + Metformin 12 cycles of weekly paclitaxel 80 mg/m^2^ + Metformin 1500 mg/day	Reduced the tumor volume by 4-fold in HER2+, trastuzumab resistant tumor	Metformin reduces the cardiotoxicity induced by trastuzumab	Metformin lowers circulating insulin-like growth factor (IGF). Inhibition of AMPK/mTOR/p70S6K1 pathway	In vivo Phase II trial (2010)	[174,175]

## Abbreviations

2-DG	2-Deoxy-D-glucose
3-BrPy	3-bromopyruvate
3-PO	3-(3-pyridinyl)-1-(4-pyridinyl)-2-propen-1-one
6-AN	6-aminonicotinamide
AKT	Protein kinase B
AMPK	5’ adenosine monophosphate-activated protein kinase
ASCT2	Alanine-, serine-, cysteine transporter 2
ATO	Arsenic trioxide
ATP	Adenosine triphosphate
BC	Breast cancer
BSO	Buthionine sulfoximine
CHC	α-cyano-4-hydroxycinnamate
DCA	Dichloroacetate
ECM	Extracellular matrix
EGFR	Epidermal growth factor receptor
EN	Enolase
ER	Estrogen receptor
GAPDH	Glyceraldehyde-3-phosphate dehydrogenase
GLUT	Glucose transporter
HIF-1α	Hypoxia inducible factor alpha
HK	Hexokinase
HSF1	Heat shock factor 1
IC_50_	Half maximal inhibitory concentration
IGFR	Insulin-like growth factor receptor
LDHA	Lactate Dehydrogenase A
MCP	Mitochondrial pyruvate carrier
MCT1	Monocarboxylate transporter 1
MDR	Multidrug resistance
MET	Mesenchymal-epithelial transition factor
OCT4	Octamer binding transcription factor/protein 4
P-gp	Permeability glycoprotein
PAMAM-PTX	Paclitaxel-conjugated polyamidoamine
PDTC	Pyrrolidine dithiocarbamate
PFK	Phosphofructokinase
PFKFB3	6-phosphofructo-2-kinase/fructose-2, 6-bisphosphatase 3
PI3K	Phosphatidylinositol 3-kinase
PK	Pyruvate kinase
PPP	Pentose phosphate pathway
Ras	Rat sarcoma viral proto-oncogene
ROS	Reactive oxygen species
RTK	Receptor tyrosine kinase
SOX2	Sex determining region Y Box 2
TCA	Tricarboxylic acid cycle
TNBC	Triple negative breast cancer
UA	Ursolic acid
VEGFα	Vascular endothelial growth factor alpha
WT	Wildtype
